# The Hygroscopic Opening of Sesame Fruits Is Induced by a Functionally Graded Pericarp Architecture

**DOI:** 10.3389/fpls.2016.01501

**Published:** 2016-10-10

**Authors:** Ilana Shtein, Rivka Elbaum, Benny Bar-On

**Affiliations:** ^1^Department of Mechanical Engineering, Ben-Gurion University of the NegevBeer-Sheva, Israel; ^2^The Robert H. Smith Institute of Plant Sciences and Genetics in Agriculture, The Hebrew University of JerusalemRehovot, Israel

**Keywords:** hygroscopic movement, mechanical model, *Sesamum indicum*, capsule, biomechanics, biocomposite

## Abstract

To enhance the distribution of their seeds, plants often utilize hygroscopic deformations that actuate dispersal mechanisms. Such movements are based on desiccation-induced shrinkage of tissues in predefined directions. The basic hygroscopic deformations are typically actuated by a bi-layer configuration, in which shrinking of an active tissue layer is resisted by a stiff layer, generating a set of basic movements including bending, coiling, and twisting. In this study, we investigate a new type of functionally graded hygroscopic movement in the fruit (capsule) of sesame (*Sesamum indicum* L.). Microscopic observations of the capsules showed that the inner stiff endocarp layer is built of a bilayer of transverse (i.e., circumferential) and longitudinal fiber cells with the layers positioned in a semi-circle, one inside the other. The outer mesocarp layer is made of soft parenchyma cells. The thickness of the fibrous layers and of the mesocarp exhibits a graded architecture, with gradual changes in their thickness around the capsule circumference. The cellulose microfibrils in the fiber cell walls are lying parallel to the cell long axis, rendering them stiff. The outer mesocarp layer contracted by 300% as it dried. Removal of this outer layer inhibited the opening movement, indicating that it is the active tissue. A biomechanical hygro-elastic model based on the relative thicknesses of the layers successfully simulated the opening curvature. Our findings suggest that the sesame capsules possess a functionally graded architecture, which promotes a non-uniform double-curvature hygroscopic bending movement. In contrast to other hygroscopic organs described in the literature, the sesame capsule actuating and resisting tissues are not uniform throughout the device, but changing gradually. This newly described mechanism can be exploited in bio-inspired designs of novel actuating platforms.

## Introduction

As plants are sessile organisms, effective seed dispersal is a critical factor in their survival. Plants utilize several intricate movement mechanisms for seed dispersal, while hygroscopic (humidity-driven) movements are very common. Such hygroscopic movements are generated upon shrinking or swelling of an organ in predefined directions, and subsequently causing a change in its structural configuration (Burgert and Fratzl, 2009b; Bar-On et al., 2014; Elbaum and Abraham, 2014). Plant tissues are composed of cells with rigid walls, viewed as a composite material, made of stiff cellulose microfibrils embedded in a soft bio-polymeric matrix, and the architecture of which dominates various biomechanical properties (Burgert and Fratzl, 2009b). Macroscopic movement is governed both by the cellulose microfibrils arrangement and the tissue architecture (the tissue types and the general morphology of the organ) (Niklas, 1992). Several types of hygroscopic movement mechanisms can be identified, always involving a layered tissue structure with distinctive hygro-elastic characteristics. The simplest type involves a pure bending, originating from a layered architecture with tissue orientation aligned with the organ axis [e.g., wild wheat (*Triticum dicoccoides*) awns (Elbaum et al., 2007), pine cone scales (Dawson et al., 1997), carrot flowers (Lacey et al., 1983), geranium awns (Abraham and Elbaum, 2013a), and *Jacaranda mimosifolia* pods (Bar-On et al., 2014)]. A more intricate type, exhibiting coupled bending and twisting deformations, emerges when the tissue layers orientation is not aligned with the organ axis [*Bauhinia variegata* pods (Armon et al., 2011), *Leucaena leucocephala*, and *Erythrina corallodendrum* fruits (Bar-On et al., 2014), several members of the pea family pods (Fahn and Zohary, 1955)].

**FIGURE 1:**
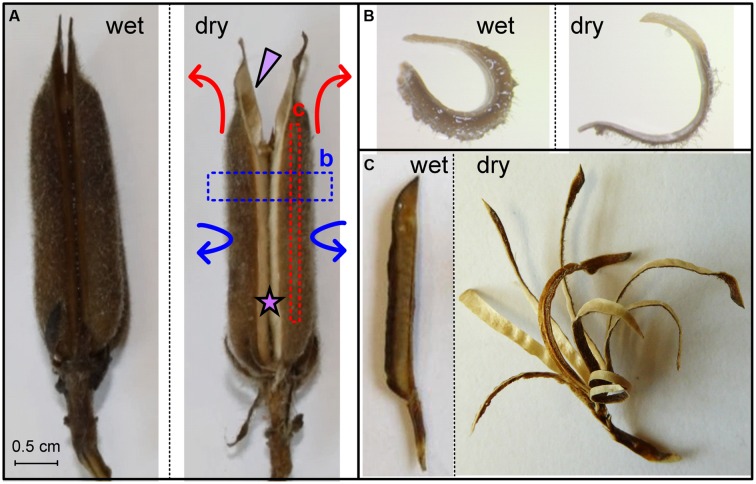
**Sesame capsule hygroscopic movement. (A)** Movement of the whole capsule. The dry capsule opens along a split region, which is marked by a star. An arrowhead points to one of four seed-containing locules. Arrows represent the circumferential (blue) and longitudinal (red) movements. **(B)** Transverse section, marked by a blue box in **(A)**, is straightening upon drying. **(C)** Longitudinal segments are marked by a red box in **(A)**. Note the bending gradient along the locule circumference. Scale bars represent 5 mm.

Sesame (*Sesamum indicum* L.) fruits are structured as cylindrical capsules, rather than planar pods or uniaxial awns, and exhibit a fundamentally different hygroscopic movement than the types studied so far (**Figure 1A**). A number of studies attempted to characterize the morphology and material architecture of the sesame capsules, and its structure was reported to consist of four or eight seed compartments (locules) with an outer soft parenchymatous mesocarp layer and an inner stiff fibrous endocarp (Ashri and Ladijinski, 1964; Day, 2000a,b). Accordingly, the hygroscopic opening of the capsule was assumed to originate from differential desiccation and shrinking ratios of the layers (parenchyma vs. fibers). These previous works, however, focused only on the initial fracture of the sesame capsule and disregarded the subsequent hygroscopic deformations that follow.

In this work, we thoroughly explore the structure of the individual layers composing the capsule, including their local geometrical parameters and the cellulose microfibril directionality, and propose a biomechanical modeling framework for the hygroscopic movement. We demonstrate that the sesame capsules represent a functionally graded hygroscopic movement.

## Materials and Methods

### Plant Material

Mature and dry *S. indicum* cv. S-327 capsules were kept under ambient conditions. Prior to all experiments, the capsules were rehydrated for 24 h in a plastic tube with tap water.

### Histology

Plant material was fixed in FAA (5:5:90, formalin: acetic acid: 70% ethanol), dehydrated in a graded ethanol solution series and then embedded in paraffin wax. Sections of 12 μm thickness were cut with a rotary microtome (Leica RM2255, Leica Biosystems, Germany) and stained with Toluidine Blue O (TBO; O’Brien et al., 1964). For differential staining of primary and secondary cell walls, the sections were stained either with Alcian Green-Safranin (Joel, 1983) or Safranin-Fast Green (Ruzin, 1999). Hand sections were made with disposable razor blades. The sections were viewed and photographed under a stereo microscope (Olympus SZ2-ILST) equipped with a camera (Olympus LC20).

### Anatomical Parameters

Cell size parameters were measured with ImageJ software^1^ [Rasband, W.S., ImageJ, U. S. National Institutes of Health, Bethesda, MD, USA, 1997–2015, (Schneider et al., 2012)]. The circumference of each locule was measured and 20 points were marked with equal distances between them. Mesocarp parenchyma thickness (X_M_), longitudinal fiber thickness (X_L_), and transverse fiber thickness (X_T_) were measured at each point.

### Estimating Cellulose Microfibrils Angle (MFA) by Polarized Light Microscopy

Crystalline cellulose is a strongly birefringent material. LC-PolScope image processing system (CRi, Inc., Woburn, MA, USA) with liquid crystal (LC) compensator enables the assessment of the sample light retardance, from which cellulose microfibril angles can be estimated (Abraham and Elbaum, 2013b). Images of 12 μm thickness sections were taken using a microscope (Nicon Eclipse 80i) under Pan Flour x20/0.5 objective, with retardance range of 0–135 nm. Image analysis was done using Abrio 2.2 software (CRi, Inc., Woburn, MA, USA). In each sample, at least five cells of longitudinal and five cells of transverse fiber cell types were measured, with three measurements per cell.

### Curvature Measurements

Curvature measurements were done after Chen et al. (2011). Rehydrated capsules were divided into locules. For curvature measurements, three locules from three different capsules were carefully cut into 8–9 longitudinal segments with nail scissors. After dehydration and subsequent bending, the segments were removed (keeping their order) and photographed (Supplementary Figure S1). Using ImageJ software, each segment was fitted to a circle. The radius (R) of a circle (for each segment) and the width (d_n_) of each segment were measured with ImageJ software. The curvature along each segment was calculated as κ = 1/R. The relative position on the circumference for the nth segment (of total N segments) was calculated via $l_{n}=\Sigma_{1}^{n}\,d_{i}/\Sigma_{1}^{N}\,d_{i}$.

### Structural-Mechanical Modeling

A theoretical mechanics of composite materials model (Hull, 1981; Gibson, 1994) was developed to analyze the correlation between the local material-level architecture and the dehydration-induced deformations in longitudinal segments of sesame capsules (see **Figure 1C**; Supplementary Figures S1 and S2). The capsule wall was viewed as a multi-layer structure, for which an analytical relation between the hygroscopic shrinkage/expansion of the layers (ε) and the longitudinal curvature change of the capsule wall (k_L_) was formulated as κ_L_ = f*ε*. The proportion coefficient (fε) is an explicit function of the stiffness anisotropy, thickness, and sequence of the individual layers (see Supporting Information for a more detailed explanation of the model). Similar methodology was recently applied to analyze the shape change of plant tissues – and was found to provide adequate predictions for the gradual change in the deformation patterns upon various types of seed pods upon hydrating/dehydrating (Bar-On et al., 2014).

Following fundamental mechanics of materials models [direct and inverse rule of mixtures, the Voigt and Reuss models, respectively (Hull, 1981)], we assumed that the stiffness of the fiber layers along the cell axis is dominated by the modulus of the microfibrils. The stiffness of the fiber layers in the perpendicular direction and the isotropic stiffness of the parenchyma layer are dominated by the modulus of the non-cellulosic matrix. A stiffness ratio of 20 (along the fibers) to 5 (perpendicular to the fibers) to 0.1 (parenchymatous mesocarp) was selected to express these considerations, based on typical values from the literature (Gibson, 2012; Bar-On et al., 2014).

## Results

### Description of the Opening Movement

Sesame seeds are located in prolonged and grooved capsules (**Figure 1A**). Each capsule consists of four long round locules (seed compartments) containing rows of seeds. As the capsule matures, it dries, splits along an abscission tissue, and hygroscopically opens, allowing the seeds to disperse. The hygroscopic opening movement is complex, simultaneously bending backward and sideward as indicated by arrows in **Figure 1A**. The opening movement was reversible, namely, rehydration closed the capsule, and subsequent drying reopened it.

To investigate further the movement, we cut excised rehydrated capsule locules into segments both parallel and perpendicular to the capsule’s long axis. Transverse sections (perpendicular to the capsule’s long axis) straightened upon drying (**Figure 1B**), with serial transverse sections along the length of the capsule exhibiting similar bending pattern. Longitudinal segments (parallel to the capsule’s long axis) bent outward as they dried (**Figure 1C**). We found a gradient in the curvature of the longitudinal cuts: minimal bending was observed at the split region; the bending curvature increased along the circumference of the seed compartment (locule) to a maximum just before the main vascular bundle and after that reduced abruptly (Supplementary Figure S1). We want to emphasize, that longitudinally a sesame capsule is quite uniform in its structure- therefore subsequent transverse sections have the same structure and thus behavior. Cross (transverse) section of a locule always shows a movement shown in **Figure 1B**. Longitudinal sections, on the other hand, have a different structure- each longitudinally serially excised segment has a different proportion of fibers and mesocarp, being located on a different circumferential point. Thus they show a bending gradient around the capsule locule, as shown in **Figure 1C**.

### Analysis of the Capsule Structure and Movement

**FIGURE 2:**
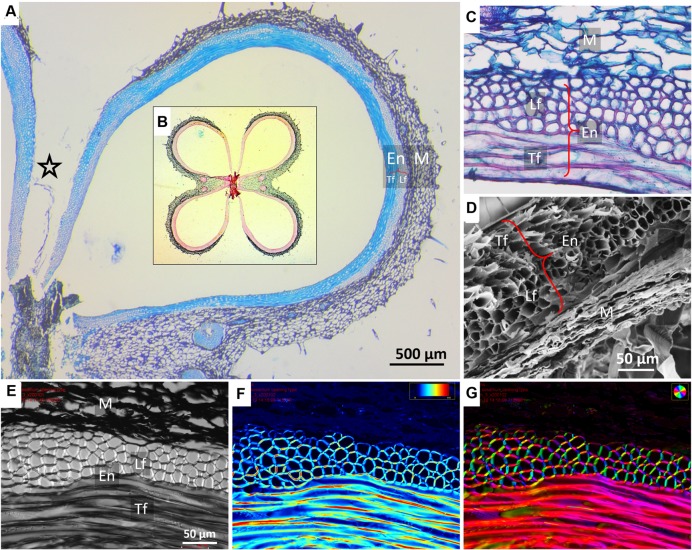
**Microscopic structure of a sesame capsule in transverse sections. (A)** Cross section of a capsule locule stained by TBO, demonstrating the variation in the relative thicknesses of the pericarp layers. A star marks the split region. Insert **(B)**: an overview of a capsule cross section showing all four locules stained by Safranin–Alcian blue. Lignified fibers stained red, cellulose bluish-green. **(C)** A close-up on the layers in the cross section, safranin-fast green staining. **(D)** Scanning electron micrograph of the layers, showing a contracted dried mesocarp (parenchyma) tissue. **(E–G)** Polarized light micrographs of a capsule cross section showing **(E)** a general view, **(F)** light retardance map (scale, 0–191 nm retardation), and **(G)** crystalline cellulose microfibrils orientation map. The orientation is color-coded. Note that the mesocarp presents much smaller light retardance than the fibers, and therefore it appears dark in panels **(F,G)**. Notations: M, parenchyma tissue; En, endocarp; Lf, longitudinal fibers; Tf, transverse fibers (circumferential direction).

**FIGURE 3:**
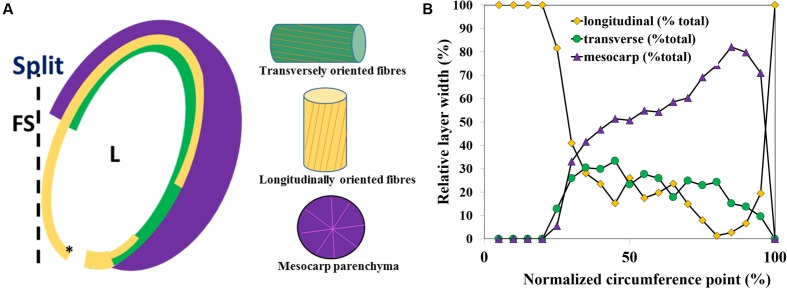
**Capsule locule geometry along the locule circumference in a transverse section. (A)** Schematic representation of the layered structure of a single capsule locule. **(B)** A plot of the relative thickness (x) of the parenchyma (mesocarp), and the endocarp longitudinal and transverse fiber layers width along the locule circumference, measured from the split region. L, locule; FS, false septum; the asterisk shows the 0 circumference point.

The sesame capsule exhibits a layered structure, comprised of outer soft layer (mesocarp) and inner stiff layer (endocarp) (**Figures 2** and **3A**). The outer layer is made of parenchyma, which is a plant tissue type, characterized by thin-walled isotropic cells. The inner layer consists of a bilayer of long and narrow fiber cells, directed in transverse and longitudinal orientations with respect to the capsule long axis. The longitudinally oriented fiber cells are positioned on the outer side of the fiber layer, while the transversely oriented fibers are positioned on the inside. In both fiber layers polarized light microscopy showed that the crystalline cellulose microfibrils lie almost parallel to the fiber cells’ axis, rendering the cells relatively stiff (**Figures 2F,G**). The microfibril angles relative to the cell’s long axis were 8 ± 0.6° in the transverse cells, and 12 ± 2° in the longitudinal cells.

The relative thickness of the sublayers was found to change gradually along the circumference of the capsule (**Figure 3**). Near the split region there were only longitudinally oriented fiber cells (See **Figures 2A** and **3**). The relative proportion of longitudinal fibers reduced gradually, with increasing parenchyma and transverse fiber tissues. Mostly parenchyma and transverse fiber cells were found near the vascular bundle, and next to it, at the farthermost point from the split, mostly longitudinal cells were found (**Figures 2A** and **3**). Interestingly, a strong reverse correlation was found between the relative thickness of the parenchyma and the layer of the longitudinal fiber cells (*R*^2^= 0.946), but not to that of transverse fiber cells (*R*^2^= 0.416). This morphological correlation suggested that the parenchyma has a role in the capsule hygroscopic opening.

**FIGURE 4:**
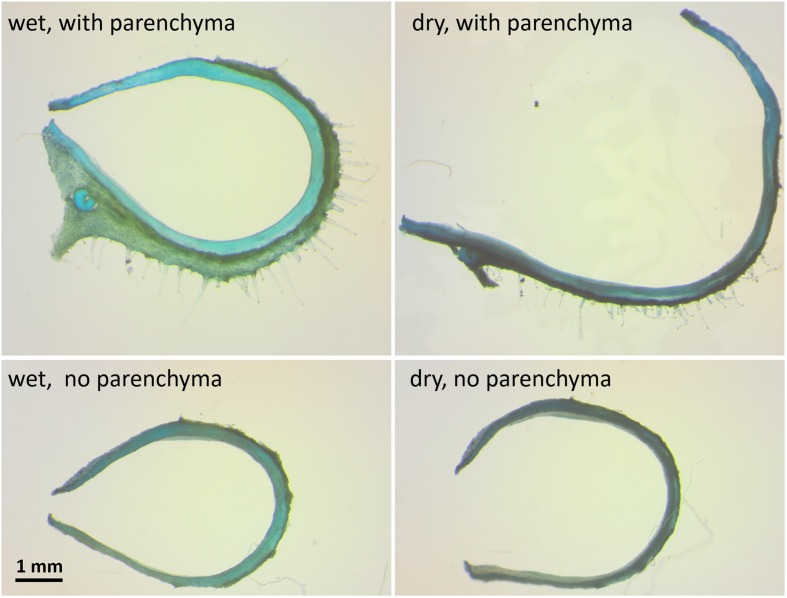
**The influence of the mesocarp parenchyma layer on sesame capsule hygroscopic movement**. Transverse sections of a capsule before and after the outer mesocarp layer was removed, stained by TBO. The hygroscopic movement of the segment is inhibited with parenchyma removal. Note the large change in the mesocarp thickness between the wet and the dry state in the upper panels.

To test whether the parenchyma is dominant in the hygroscopic movement, we sectioned a hydrated capsule transversely As the sections dried, they straightened, with the parenchyma shrinking to 30% of its wet-state thickness (**Figures 1B** and **4**). The same experiment was conducted after the outer parenchyma layer was carefully removed. As the section dried, the dehydration induced hygroscopic movement was substantially inhibited (**Figure 4**).

### Bio-mechanical Modeling

A multi-scale structural-mechanical model was developed to gain insights on the key parameters that dominate capsules motion. As an input, the model used the local layered architecture of the capsule wall, and calculated the longitudinal bending curvature at each circumferential location (as shown in Supplementary Figure S1). To proceed with an adequate, but simplified, mechanical modeling, the parenchyma tissue was considered as a quasi-isotropic material while each of the fiber layers was considered as anisotropic parallel-fiber composite materials. Following the experimental observations that the parenchyma exhibited considerable hygroscopic expansion/shrinkage (∼300% change, **Figures 1B** and **4**) while for the fiber layers these effects were found to be comparatively negligible- only the hygroscopic expansion/shrinkage of the parenchyma layer were considered in our modeling. It should be noted that the proposed simplified model does not intend to provide accurate quantitative predictions, but to capture the essence of the hydration/dehydration induced deformations phenomenon in the sesame capsules.

**FIGURE 5:**
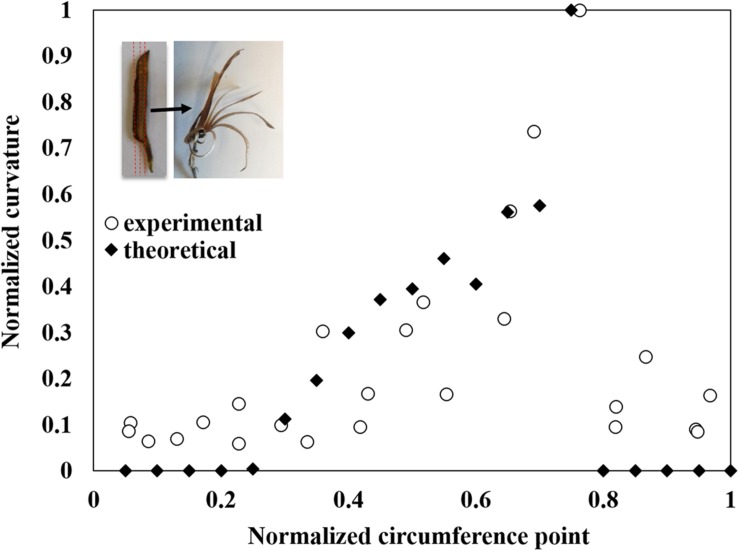
**Modeling of the capsule’s hygroscopic movement**. The measured longitudinal bending curvature normalized to the maximal curvature between all segments along the locule circumference (see **Figure 1C** and Supplementary Figure S1) is compared to theoretically predicted values. The movement concentrated where the transverse fibers create a monolayer, at about 75% along the section (measured from the split region). The insert shows the direction of the segmentation and the following hygroscopic curvature.

Once the effective elastic and hygroscopic characteristics of the individual layers in the sesame capsule were defined, a macroscopic laminate-mechanics model was formulated to evaluate the local bending curvature in the capsule wall – by considering the explicit thickness and sequence of the layer across the capsule – which gradually varies with the capsule circumference. The resultant theoretical predictions are in a very good agreement with the independent experimental observations (**Figure 5**).

## Discussion

Hygroscopic movement requires a carefully orchestrated array of physiological, anatomical and hygro-elastic factors. Hygroscopic movements in plants typically originate from either anisotropic shrinkage of the fibrous layers according to their cellulose orientation [e.g., pine cones (Dawson et al., 1997), wheat awns (Elbaum et al., 2007)], or isotropic shrinkage of a parenchyma layer, resisted by the fibrous layers [described in pods of *L. leucocephala*, *J. mimosifolia*, and *E. corallodendrum*, (Bar-On et al., 2014), flower petal movement in *Syngonanthus elegans* (Oriani and Scatena, 2009), opening movement of *Lilium* and *Arabidopsis* anthers (Nelson et al., 2012), or movement of *Daucus carota* umbellet peduncles (Lacey et al., 1983)]. Simple histology of sesame capsules reveals both structural configurations, and thus, on a first sight, both mechanisms can potentially lead to capsule opening.

Our findings indicate that the opening movement is driven by the shrinkage of parenchyma layer relative to the fiber layers. Previous works (Ashri and Ladijinski, 1964; Day, 2000a,b) suggested that this differential shrinking of the layers resulted in the initial breakage in the weakest part of the capsule – the false septa – and attempted to measure these fracture forces (Day, 2000b). In contrast, we studied the post-fracture hygroscopic deformations of the capsule, dominated by the underlying cell wall structure and the complex morphology of the organ that set the intrinsic biomechanical properties of the material.

In a mechanics-of-materials perspective, plant cell walls can be viewed as a bio-composite material of highly stiff cellulose microfibrils embedded in a much more compliant non-cellulosic matrix phase. Despite the similarity at the composition level, the parenchyma and fiber layers exhibit very different multi-scale material architectures that prompt large differences in their mechanical characteristics. The parenchyma is structured as a foam, with no evidence for a preferred microfibril direction in its cell walls. Contrary to this, each of the two fiber sublayers includes a well defined array of unidirectional tubular fiber cells, with microfibril angles almost parallel to the longitudinal cell axis of the fibers (∼10°) (**Figure 2**). The two fiber sublayers are oriented perpendicularly to each other, forming a plywood structure. Upon drying, the fibers resist movement along them, thus, the longitudinally oriented fibers would enable a transverse outward bending movement and the transversally oriented fibers- a longitudinal bending. Each fiber layer resists the movement in the other direction. In comparison, in *B. variegata* pods hygroscopic opening movement is directed by a fiber bilayer, with layers oriented at ± 45° to the pod long axis (Armon et al., 2011). However, in sesame capsules the fibers are not the actuator of the movement. Three indications suggest that the parenchyma shrinkage mechanisms dominated the sesame opening, where the anisotropic bilayer shrinkage had only a minor effect: (1) The parenchyma cells shrunk considerably while drying (300% change between rehydrated versus dry capsules). (2) The hygroscopic movement was almost eliminated in cross sections of the capsule when the parenchyma was removed (**Figure 4**). (3) The strongest longitudinal bending occurred in the monolayer area of longitudinal fibers (compare **Figures 3** and **5**). This suggests that the transverse fibers are not necessary for the longitudinal bending. The transverse fibers most likely have an equivalent role in the capsule opening along the transverse direction (**Figures 1B** and **4**), and might serve in capsule enforcement promoting its structural stability.

Perhaps the unique finding of this work is that in contrast to other studied hygroscopically active tissues, the sesame capsule actuating tissues are not uniform throughout the device, but changing gradually. The opening of the sesame capsule involves a bi-axial motion with simultaneously bending outward in longitudinal and transverse directions (**Figure 1**). The complex movement that opens the dehiscent capsule combines the two orthogonal movements, which are enabled both by strong shrinking abilities of the outer parenchymatous layer, as well as the gradual changes in the relative thickness of the three tissue layers. An analytical hygro-elastic model, based only on the thicknesses of the active and resistance layers, was found to be in a good agreement with the experimental results for the localized longitudinal bending curvatures at different circumferential locations. In the model, the fiber shrinking was neglected and only the parenchyma was assumed to change its dimensions. Thus, it further supports the conclusion that a functionally graded layered construct is the dominant effect in the elaborate hygroscopic movement of the sesame capsule. Employing these material-level principles into synthetic analogs may potentially pave the way to the development of new types of actuation systems with multi-axis graded shape morphing capabilities (Burgert and Fratzl, 2009a; Erb et al., 2013; Bar-On et al., 2014).

The hygroscopic opening of the sesame capsule was found to originate from an active parenchyma layer resisted in two orthogonal directions by an inner fibrous bi-layer. Shrinkage of the parenchyma produces a graded spatial distortion of the cylindrical manifold via a bi-axial bending. The non-uniform relative thickness of the layers promotes a graded bi-axial bending, leading to the complex capsule opening movement.

## Author Contributions

IS design of the work, acquisition, and analysis of data, writing; RE design of the work and analysis of data; BB-O design of the work, acquisition, and analysis of data.

## Conflict of Interest Statement

The authors declare that the research was conducted in the absence of any commercial or financial relationships that could be construed as a potential conflict of interest.

## Footnotes

1 http://imagej.nih.gov/ij/
