# Supplementary material for: The Hygroscopic Opening of Sesame Fruits Is Induced by a Functionally Graded Pericarp Architecture
Source: Front Plant Sci. 2016 Oct 10;7:1501. doi: 10.3389/fpls.2016.01501 (PMC5056167; doi:10.3389/fpls.2016.01501)
Supplement: Supplementary file 1 [file Data_Sheet_1.PDF]

Supporting Information

The Hygroscopic Opening of Sesame Fruits is Induced by a Functionally Graded Pericarp Architecture

Ilana Shtein[1], Rivka Elbaum[2], Benny Bar-On[1*]

[1]Department of Mechanical Engineering, Ben-Gurion University of the Negev, Beer-Sheva, 84105, Israel

[2]The Robert H. Smith Institute of Plant Sciences and Genetics in Agriculture, The Hebrew University of Jerusalem, Rehovot, Israel

Longitudinal segments of the sesame capsule and their bending curvatures

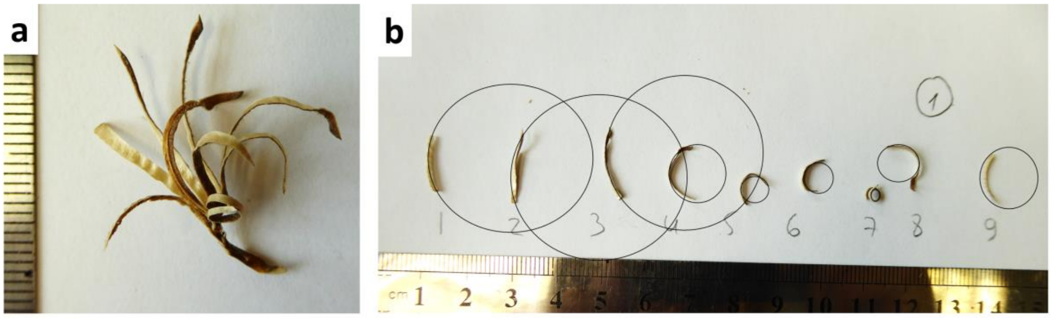

***Supplementary Figure S1*: Quantification of hygroscopic movement in the longitudinal plane. (a) Capsule locules were sectioned longitudinally in the wet state into eight/nine segments and allowed to dry. (b) The segments were carefully removed for curvature and thickness measurements. Note the bending gradient along the locule circumference. Scale ticks represent 1 mm.**

Hygro-mechanical model for the longitudinal bending curvatures

In this supporting information section we briefly draw the basic principles used for the theoretical model used for analysing the longitudinal deformation curvatures of the sesame capsule. A comprehensive description of the guiding biomechanical principles of our model can be found in standard textbooks on composite mechanics (e.g. Hull 1981, Gibson 1994).

We consider a longitudinal segment of the capsule (shown in Supplementary Figure 2) composed of three layers: longitudinal fibers (L), transverse fibers (T) and mesocarp (M). The individual thickness of these layers is indicated by X_L, X_T and X_M, and the distance of the inner face and the individual layer edges from the centreline are indicated by h_0, h_L, h_T and h_M, respectively.

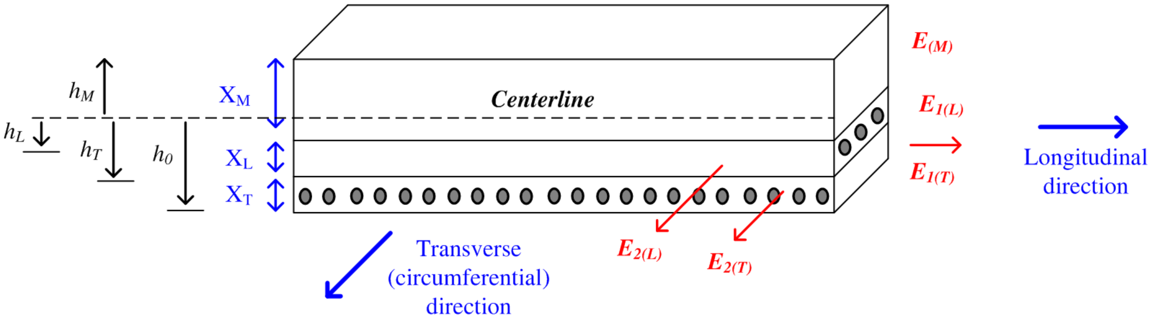

***Supplementary Figure 2**: Schematic description of a longitudinal segment from the sesame capsule with the related geometrical and mechanical parameters.*

The mesocarp is considered as an isotropic material, characterized by Young's modulus ($E_{(M)}$) and a Poisson's ratio ($\nu_{(M)}$). The longitudinal and transverse fibrous layers are considered as anisotropic materials, characterized by longitudinal and transverse moduli ($E_{1(L)}$ and $E_{1(T)}$; $E_{2(L)}$ and $E_{2(T)}$), major Poisson ratios ($\nu_{12(L)}$ and $\nu_{12(T)}$) and shear moduli ($G_{12(L)}$ and $G_{12(T)}$).

Upon hygroscopic deformations of the mesocarp layer by a strain of ε, the capsule walls experience pseudo bi-axial hygroscopic forces moments at the longitudinal (L) and transverse (T) directions:

$$F = \left[E_{(M)}\left(1+\nu_{(M)}\right)/\left(1-\nu_{(M)}^2\right)\right] \cdot (h_M - h_L) \cdot \varepsilon \qquad (1a)$$

$$M = \left[E_{(M)}\left(1+\nu_{(M)}\right)/\left(1-\nu_{(M)}^2\right)\right] \cdot (h_M^2 - h_L^2) \cdot \varepsilon \qquad (1b)$$

The pseudo hygroscopic forces and moments result in both in-plane strains and out-of-plane curvatures that are related through the laminate stiffness matrix:

$$\bar{P} = \boldsymbol{K} \cdot \bar{\delta} \qquad (2)$$

Where $\bar{P}$ and $\bar{\delta}$ are the generalized hygroscopic loads and deformations vectors:

$$\bar{P} = [F, F, 0, M, M, 0]^T = \bar{p} \cdot \varepsilon \qquad (3a)$$

$$\bar{\delta} = [\varepsilon_L, \varepsilon_T, 0, \kappa_L, \kappa_L, 0]^T \qquad (3b)$$

With ε_L and ε_T are the in-plane strains at the longitudinal and transverse directions, and κ_L and κ_T are the bending curvatures at the longitudinal and transverse directions. $\bar{p}$ is a pseudo generalized load vector per unit hygroscopic strain, as extracted from eqs. 1(a-b). Note that the zeros at the generalized loads and deformations vectors represent zero shearing and twisting forces and deformations since the hygroscopic deformations emerges at the principal axial of the capsule (i.e. along the longitudinal and transverse fibril directions).

The stiffness matrix $\boldsymbol{K}$ (6×6) in eq.(2) is a composition of three sub-matrixes $\boldsymbol{A}, \boldsymbol{B}, \boldsymbol{D}(3 \times 3)$ as follows:

$$\boldsymbol{K} = \begin{bmatrix} \boldsymbol{A} & \boldsymbol{B} \\ \boldsymbol{B} & \boldsymbol{D} \end{bmatrix} \tag{4}$$

Where $\boldsymbol{A}$ represent the in-plane stiffness of the layered material (forces-strains), $\boldsymbol{D}$ represent the out-of-plane stiffness (moments-curvatures), and $\boldsymbol{B}$ represent the coupling in-plane out-of-plane stiffness (forces-curvatures, moments-strains).

The $\boldsymbol{A}$, $\boldsymbol{B}$ and $\boldsymbol{D}$ matrixes are related to the stiffness matrixes (3×3) of the individual longitudinal, transverse and mesocarp layers (i.e. $\boldsymbol{Q}_{(L)}$, $\boldsymbol{Q}_{(T)}$ and $\boldsymbol{Q}_{(M)}$) and their dimensions via:

$$\boldsymbol{A} = \boldsymbol{Q}_{(T)}(h_T - h_0) + \boldsymbol{Q}_{(L)}(h_L - h_T) + \boldsymbol{Q}_{(M)}(h_M - h_L) \tag{5a}$$

$$\boldsymbol{B} = \frac{1}{2}\left[\boldsymbol{Q}_{(T)}(h_T^2 - h_0^2) + \boldsymbol{Q}_{(L)}(h_L^2 - h_T^2) + \boldsymbol{Q}_{(M)}(h_M^2 - h_L^2)\right] \tag{5b}$$

$$\boldsymbol{D} = \frac{1}{3}\left[\boldsymbol{Q}_{(T)}(h_T^3 - h_0^3) + \boldsymbol{Q}_{(L)}(h_L^3 - h_T^3) + \boldsymbol{Q}_{(M)}(h_M^3 - h_L^3)\right] \tag{5c}$$

Where the stiffness matrix of the individual layers are defined by their moduli as follows:

$$\boldsymbol{Q}_{(L)} = \begin{bmatrix} E_{1(L)}/\left(1 - \nu_{12(L)}^2 \frac{E_{2(L)}}{E_{1(L)}}\right) & \nu_{12(L)} E_{2(L)}/\left(1 - \nu_{12(L)}^2 \frac{E_{2(L)}}{E_{1(L)}}\right) & 0 \\ & E_{2(L)}/\left(1 - \nu_{12(L)}^2 \frac{E_{2(L)}}{E_{1(L)}}\right) & 0 \\ sym & & G_{12(L)} \end{bmatrix} \tag{6a}$$

$$\boldsymbol{Q}_{(T)} = \begin{bmatrix} E_{1(T)}/\left(1 - \nu_{12(T)}^2 \frac{E_{2(T)}}{E_{1(T)}}\right) & \nu_{12(T)} E_{2(T)}/\left(1 - \nu_{12(T)}^2 \frac{E_{2(T)}}{E_{1(T)}}\right) & 0 \\ & E_{2(T)}/\left(1 - \nu_{12(T)}^2 \frac{E_{2(T)}}{E_{1(T)}}\right) & 0 \\ sym & & G_{12(T)} \end{bmatrix} \tag{6b}$$

$$\boldsymbol{Q}_{(M)} = \begin{bmatrix} E_{(M)}/\left(1 - \nu_{(M)}^2\right) & \nu_{(M)} E_{(M)}/\left(1 - \nu_{(M)}^2\right) & 0 \\ & E_{(M)}/\left(1 - \nu_{(M)}^2\right) & 0 \\ sym & & E_{(M)}/2\left(1 + \nu_{(M)}\right) \end{bmatrix} \tag{6c}$$

Note that the zeros at the $\boldsymbol{Q}_{(L)}$, $\boldsymbol{Q}_{(T)}$ and $\boldsymbol{Q}_{(M)}$matrices represent no tensile-shearing couplings since the principal directions of the individual layers coincide with the principal axes of the capsule (i.e. orthotropic materials).

By substituting eqs. (1,3,4) into eq. (2) the hygroscopic deformations of the locule can be extracted analytically - as a function of the locule geometrical and mechanical characteristics:

$$\bar{\delta} = (\boldsymbol{K}^{-1} \cdot \bar{p}) \cdot \varepsilon \qquad (7)$$

Finally the localized hygroscopic longitudinal curvatures of the capsule can thus be expressed by the mesocarp deformations and a proportion coefficient (*f*), as extracted from eqs. (3b,7):

$$\kappa_L = f \cdot \varepsilon \qquad (8)$$

References

Gibson R. F. (1994). *Principles of composite material mechanics*, Mc-Graw-Hill.

Hull, D. (1981). *An introduction to composite materials*. Cambridge: Cambridge University Press.
